# Effectiveness of vedolizumab and ustekinumab as second biologic agent in achieving target outcomes in tumor necrosis factor antagonists experienced patients with inflammatory bowel disease (enroll-ex study)

**DOI:** 10.3389/fphar.2023.1243080

**Published:** 2023-10-09

**Authors:** Fatema Alrashed, Israa Abdullah, Ahmad Alfadhli, Mohammad Shehab

**Affiliations:** ^1^ Department of Pharmacy Practice, College of Pharmacy, Kuwait University, Kuwait City, Kuwait; ^2^ Department of Internal Medicine, Mubarak Al-Kabeer University Hospital, Jabriya, Kuwait; ^3^ Department of Biochemistry and Molecular Biology, Dasman Diabetes Institute, Kuwait City, Kuwait

**Keywords:** surgery, hospitalization, steroids, endoscopic, remission, biologics, inflammatory bowel disease

## Abstract

**Background:** About a third of patients with inflammatory bowel disease (IBD) do not respond to anti-tumor necrosis factor (anti-TNF) therapy. In our study, we evaluated the effectiveness of vedolizumab and ustekinumab in achieving clinical and endoscopic outcomes in anti-TNF-experienced patients with IBD.

**Methods:** We conducted a retrospective cohort study. Electronic medical records of patients with moderate to severe IBD, who were previously received anti-TNF therapies, were reviewed and evaluated retrospectively in a gastroenterology center. Outcomes of patients treated with ustekinumab or vedolizumab after failing one anti-TNF agent were evaluated. The primary outcomes were the percentage of hospitalization, surgery, mucosal healing and steroid-free remission. Mucosal healing was defined as a Mayo endoscopic score of 0 or 1 in ulcerative colitis (UC) and an SES-CD score of less than 3 in Crohn’s disease (CD). Outcomes were quantified using descriptive analysis.

**Results:** A total of 207 (130 CD: 77 UC) patients with IBD who had previously received one anti-TNF agent were included in the study. Of the total cohort, 62 (30.0%) patients were receiving vedolizumab, and 145 (70.0%) patients were on ustekinumab. 101 (77.6%) patients with CD who failed one anti-TNF therapy were on ustekinumab. Of these patients, 26 (19.7%) patients were hospitalized, and 12 (11.9%) patients had IBD-related surgery. 16 (16.1%) patients had at least one corticosteroid course. 60 (59.0%) patients with CD on ustekinumab achieved mucosal healing. 29 (22.3%) patients with CD who failed one anti-TNF therapy were receiving vedolizumab. Of those, 7 (25%) patients were hospitalized, and 11 (37.9%) patients had IBD-related surgery. 15 (51.0%) patients achieved mucosal healing. 44 (57.1%) patients with UC who failed one anti-TNF therapy were on ustekinumab. Of these 6 (14.1%) patients were hospitalized, 3 (7.0%) patients had IBD-related surgery and 13 (30%) patients had at least 1 corticosteroid course. 25 (57.0%) patients achieved mucosal healing. 33 (42.8%) patients with UC who failed one anti-TNF therapy were receiving vedolizumab. Of those, 6 (18.6%) patients were hospitalized, and 16 (49.6%) patients had at least 1 corticosteroid course. 17 (53.2%) patients achieved mucosal healing.

**Conclusion:** Ustekinumab and vedolizumab were both effective in achieving clinical outcomes in patients with IBD after failing an anti-TNF agent. However, patients receiving ustekinumab had numerically higher percentages of reaching target outcomes than patients receiving vedolizumab. A prospective head-to-head trial is warranted to confirm these findings.

## Introduction

Inflammatory bowel disease (IBD) is a lifelong disease occurring early in life which clinically contains Crohn’s disease and ulcerative colitis. The incidence and prevalence of IBD markedly increased over the second half of the 20th century, and since the beginning of the 21st century (Guan, 2019). It is projected that in the Western world, with compounding prevalence, the number of patients with IBD will grow exponentially in the next decade. Additionally, prevalence of IBD in newly industrialized countries is a fraction of that in the Western world, but the rate of the rise in incidence is steep in newly industrialized countries. In 2025, accessibility, affordability, variation in healthcare resources and the cost of biologic agents could strain healthcare systems and exacerbate disparity of care across the world (Kaplan, 2015).

Anti-tumor necrosis factor agents (anti-TNF) have been widely used for approximately 25 years now. Their integration into clinical practice has greatly advanced the treatment of IBD. This has led to an exponential increase in the number of patients that are treated with anti-TNF therapy. However, despite their proven efficacy, a considerable number of patients on anti-TNF therapy fail to respond and of those patients who initially respond to an anti-TNF agent, some discontinue therapy because they lose their response or intolerance (Eder et al., 2015; Sochal et al., 2020). Additionally, evidence suggest that patients failing an anti-TNF agent are less likely to respond to another anti-TNF biologic (Stidham et al., 2014). The calculated annual risk of loss of infliximab response in patients was found to be 13% per patient-year (Gisbert and Panés, 2009). It is important to note that anti-TNF treatment may also be discontinued due treatment-related complications such as serious infections or intolerance (Beaugerie et al., 2020). This presents a therapeutic challenge for physicians in their daily clinical practice. It is believed that non-response to primary anti-TNF treatment is often considered indicative of a patient group that is inherently more resistant to treatment, potentially due to factors such as longer disease duration or complicated disease. In some cases, patients may have been previously exposed to anti-TNF agents and demonstrated inadequate response, leading to potential immunological challenges in achieving a satisfactory response to a second agent. These considerations highlight the complexity of managing patients who do not respond to anti-TNF treatment. Currently, there are no clear guidelines regarding the appropriate therapeutic option following the failure of anti-TNF therapy. Whether the next step should be to try other anti-TNF agents or swap current anti-TNF with a drug with different mechanism of action is not clear. Physicians must carefully assess and navigate various factors, including disease characteristics, treatment history, and potential adverse events, to make informed decisions and explore alternative therapeutic options for these patients.

Vedolizumab is a humanized gut-specific monoclonal antibody targeting the α4β7 integrin leading to the inhibition of leukocyte migration from the blood to the intestinal mucosa (Luzentales-Simpson et al., 2021). Integrins are cell surface transmembrane glycoproteins that mediate cell–cell interactions and play critical roles in immune cell signaling, and trafficking. Since integrins mediate trafficking and retention of immune cells to the gastrointestinal tract, it is not surprising that they are implicated in the pathogenesis of IBD. The substantial role integrins play in IBD led to identifying the blockade of integrins or cell adhesion molecules as a therapeutic target in IBD (Gubatan et al., 2021).

Ustekinumab is a fully human monoclonal antibody that targets the p40 subunit of interleukin-12 and interleukin-23 (Benson et al., 2011). Functionally, IL-23 plays a role in the host response to pathogens as it has been observed that mucosal inflammation is dependent on IL-23 production and the development of therapies directed against IL-23 moreover substantiates the detrimental role played by this cytokine in IBD pathogenesis (Sewell and Kaser, 2022).

Both vedolizumab and ustekinumab have proven their effectiveness in randomized controlled trials enrolling anti-TNF experienced patients (Feagan et al., 2013; Sandborn et al., 2013; Feagan et al., 2016). However, some can argue that study populations in clinical trials may not accurately represent the general IBD population, which may limit the generalizability of their results (Ha et al., 2012). Additionally, despite the availability of some real-world data regarding the effectiveness and safety of ustekinumab and vedolizumab in patients with IBD after failure of or intolerance to anti-TNF therapy (Barbieri et al., 2022; Onali et al., 2022; Rayer et al., 2022), limited evidence exists with respect to real-life data in IBD from Kuwait specifically and the middle east region generally. Therefore, the aim of this study was to assess the effectiveness of vedolizumab and ustekinumab in a real-life cohort of patients with IBD who had failed to respond to an anti-TNF agent.

## Materials and methods

### Study design and patient population

This study was a retrospective, observational study that involved a chart review of all patients with inflammatory bowel disease (IBD) who failed to respond to one anti-TNF agent. The study was conducted at a tertiary care hospital in Kuwait, Haya Alhabib Center. Enrollment period was between October 2017 to December 2022. This study was performed and reported in accordance with Strengthening the Reporting of Observational Studies in Epidemiology (STROBE) guidelines (von Elm et al., 2007).

Inclusion criteria consisted of: (Guan, 2019): age ⩾18 years; (Kaplan, 2015); previous diagnosis IBD; patients with moderate-to-severe ulcerative colitis defined as a clinical Mayo Score of 6–12, with endoscopic sub-score of 2–3; (Eder et al., 2015); patients with moderate-to-severe Crohn’s disease defined as a Crohn’s Disease Activity Index [CDAI] 220–450; or Simple Endoscopic Score for Crohn’s Disease (SES-CD) ⩾7; (Sochal et al., 2020); failure of one anti-TNF-α agent previously; (Stidham et al., 2014); treatment with ustekinumab or vedolizumab for active disease after failure of anti-TNF-α therapy; and (Gisbert and Panés, 2009) a minimum follow-up duration of 8 weeks after the induction therapy. Exclusion: pregnant females, patient who stopped using ustekinumab or vedolizumab due to allergy or intolerance, patient who failed more than one anti-TNF agent, patient with incomplete data, patient who received other immunosuppressant therapy for other conditions, e.g., rheumatological disease.

### Outcomes and definitions

The primary endpoints were percentage of hospitalization, surgery, corticosteroids courses received, and mucosal healing in patients with IBD receiving biologic therapies at week 52. Patients were considered to be on steroids if they received a course of prednisolone, budesonide or any steroidal medication 6 weeks or forward after starting the current biologic. Patients who did not receive any steroid courses after 6 weeks from starting the biologic were considered to be in steroid free remission. Mucosal healing is regarded as the total number of patients who achieved mucosal healing, defined as endoscopic Mayo score of 0 or 1 for patients with ulcerative colitis and Simple Endoscopic Score for Crohn’s Diseases (SES-CD) 0–2 for Crohn’s disease. The Duration of use is the average months of which patients have been on the current biologic. Moreover, the number of patients with surgeries is the number of patients who underwent inflammatory bowel related surgeries 6 weeks or more after starting vedolizumab or ustekinumab. Additionally, location and type of surgery were reported if patients had IBD related surgery. Hospitalization, on the other hand, is the number of patients hospitalized 6 weeks or more after starting the current biologic for an IBD related issue or complication.

In this study, diagnosis of IBD was made using the international classification of diseases (ICD-10 version:2016). Patients were considered to have IBD when they had ICD-10 K50, K50.1, K50.8, K50.9 corresponding to Crohn’s disease (CD) and ICD-10 K51, K51.0, K51.2, K51.3, K51.5, K51.8, K51.9 corresponding to ulcerative colitis (UC).

### Subgroup analysis

Subgroup analysis was conducted to quantify the number of episodes patients receiving ustekinumab or vedolizumab failed to reach the primary outcomes (number of steroid courses, IBD-related surgeries, hospitalization) during the 52 weeks period.

### Data collection

Using patient medical record, the following baseline patient data were obtained and entered into a common database: sex, age at diagnosis, body weight, duration of disease, smoking status, location, and classification of IBD, co-morbidities, previous IBD medications, previous exposure to an anti-TNF-α agent, concomitant use of corticosteroids, concomitant use of immunomodulators (thiopurines or methotrexate), reason for suspension of anti-TNF-α therapy (primary failure, or secondary failure), and information on perianal disease was also obtained. Additionally, clinical disease activity indicated by the Harvey–Bradshaw Index (HBI) score, and objective disease activity indicated by the C-reactive protein (CRP) level and endoscopic activity we also recorded. All patient information and details were de-identified so that their private information or identify may not be determined in any way.

### Ethical considerations

This study was reviewed and approved by the Ethical Review Board of the Ministry of Health of Kuwait (reference: 3616, protocol number 3678/2021) as per the updated guidelines of the Declaration of Helsinki (64th WMA General Assembly, Fortaleza, Brazil, October 2013) and of the US Federal Policy for the Protection of Human Subjects. Patients’ consents were waived.

### Statistical analysis

Statistical analyses were executed with IBM SPSS Statistics package (Version 25.0. Armonk, NY: IBM Corp). Descriptive statistics were used to calculate frequencies and central tendency, expressed as means with standard deviation (SD), median with interquartile range (IQR) and percentages. Percentages were used to express the rates of primary outcomes.

## Results

### Patients characteristics

A total of 207 patients with inflammatory bowel disease (IBD) were included in the study. Among the total cohort of patients, 130 (62.8%) patients had Crohn’s disease (CD), and 77 (37.2%) patients had ulcerative colitis (UC). In patients with CD, the mean age was 35.1 years of age, and 67 (51.5%) patients were male. The mean body mass index was 26.5 m^2^/kg, and the majority of patients were of middle eastern ethnicity 122 (94.0%). Majority of patients with CD had previously received infliximab [81 (62.5%)], while 49 (37.5%) patients received adalimumab. Additionally, most patients with CD received ustekinumab 101 (77.6%) and only 29 patients (22.3%) received vedolizumab. Median (IQR) duration of adalimumab and infliximab use in weeks were 42.2 (33.4–50.1) and 30.1 (25.9–48.3), respectively (Table 1).

**TABLE 1:** Demographic characteristics of patients with Crohn’s disease.

Variables	Total, N = 207
IBD type, *n* (%)	
Ulcerative colitis	77 (37.2%)
Crohn’s disease	130 (62.8%)
Crohn’s Disease (*n* = 130)	
Age (years), mean (SD)	35.1 (11.4)
Sex, *n* (%)act	
Male	67 (51.5%)
Female	63 (48.8%)
Ethnicity, *n* (%)	
Middle Eastern	122 (94.0%)
Others	8 (6.0%)
BMI m^2^/kg	26.5
Crohn’s disease (CD)	
L1: ileal	65 (50%)
L2: colonic	16 (12%)
L3: ileocolonic	45 (35%)
L4: upper gastrointestinal	4 (3%)
P: perianal^a^	30 (23%)
B1: inflammatory	59 (45%)
B2: structuring	32 (25%)
B3: penetrating	39 (30%)
Co-morbidities	
Diabetes	7 (5.3%)
Osteoarthritis	5 (3.8%)
Hypertension	3 (2.3%)
Cardiovascular Disease	4 (3.0%)
Asthma	12 (9.2%)
Laboratory tests (mean)	
CRP, mg/L	16.9
Stool fecal calprotectin, mcg/g	275
Albumin, g/L	43
Current Biologics *n* (%)	
Vedolizumab	29 (22.3%)
Ustekinumab	101 (77.6%)
Previous Biologics *n* (%)	
Adalimumab	49 (37.5%)
Infliximab	81 (62.5%)
Concomitant immunomodulator use^a^	40 (31.0%)
Previous medications *n* (%)	
Immunomodulators	35 (26.9%)
Azathioprine	18 (51.4%)
Methotrexate	10 (28.5%)
6-mercaptopurines	7 (20.1%)
No washout period^b^	88%
Duration of use (months), median (IQR)	
Adalimumab	42.2 (33.4–50.1)
Infliximab	30.1 (25.9–48.3)
Time from diagnosis^c^ (months), median (IQR)	
Vedolizumab	48.7 (33.1–55.7)
Ustekinumab	50.4 (46.1–58.3)
Duration of use/time to failure (months), median (IQR)	
Adalimumab (*n* = 49)	46.2 (30.4–51.3)
Primary non-response	4 (8.2%)
Secondary	45 (91.8%)
Infliximab (*n* = 81)	30.1 (23.9–46.3)
Primary non-response	14 (17.3%)
Secondary non-response	67 (82.7%)

^a^ Perianal disease is part of the L1-L4 location classification, not a separate subtype.

^b^ The wash-out period is defined as the time between the discontinuation of one biologic and the initiation of a second biologic.

^c^ From diagnosis to initiation of ustekinumab or vedolizumab

In patients with UC, the mean age was 33.9 years of age, and 39 (50.7%) patients were males. The mean body mass index was 25.9 m^2^/kg, and the majority of patients were of middle eastern ethnicity 69 (90.0%). Majority of patients with UC had previously received infliximab [42 (54.5%)], while 35 (45.5%) patients received adalimumab. Additionally, most patients with UC received ustekinumab 44 (57.1%) patients and 33 (42.8%) patients received vedolizumab. Median (IQR) duration of adalimumab and infliximab use in weeks were 45.2 (30.4–51.3) and 29.1 (23.9–46.3), respectively (Table 2).

**TABLE 2:** Demographic characteristics of patients with ulcerative colitis.

Ulcerative colitis (*n* = 77)	
Age (years), mean (SD)	33.9 (11.4)
Sex, *n* (%)	
Male	38 (49.3%)
Female	39 (50.7%)
Ethnicity, *n* (%)	
Middle-Eastern	69 (90.0%)
Others	8 (10.0%)
BMI m^2^/kg	25.9
Ulcerative colitis (UC)	
E1: ulcerative proctitis	14 (18%)
E2: left sided colitis	25 (33%)
E3: extensive colitis	38 (49%)
Co-morbidities	
Diabetes	6 (7.5%)
Osteoarthritis	4 (5.2%)
Hypertension	4 (5.2%)
Cardiovascular Disease	2 (2.5%)
Asthma	10 (12.8%)
Laboratory tests (mean)	
CRP, mg/L	16.1
Stool fecal calprotectin, mcg/g	274
Albumin, g/L	40
Current Biologics *n* (%)	
Vedolizumab	33 (42.8%)
Ustekinumab	44 (57.1%)
Previous Biologics *n* (%)	
Adalimumab	35 (45.5%)
Infliximab	42 (54.5%)
Concomitant immunomodulator use^a^	28 (36%)
Previous medications *n* (%)	
5-aminosalicylates	46 (60%)
Immunomodulators	17 (22%)
No washout period^a^	90%
Duration of use (months), median (IQR)	
Adalimumab	45.2 (30.4–51.3)
Infliximab	29.1 (23.9–46.3)
Time from diagnosis^b^ (months), median (IQR)	
Vedolizumab	48.7 (33.1–55.7)
Ustekinumab	50.4 (46.1–58.3)
Duration of use/time to failure (months), median (IQR)	
Adalimumab (*n* = 35)	46.2 (30.4–51.3)
Primary non-response	3 (8.6%)
Secondary non-response	32 (91.4%)
Infliximab (42)	30.1 (23.9–46.3)
Primary non-response	8 (19.0%)
Secondary non-response	34 (81.0%)

^a^ The wash-out period is defined as the time between the discontinuation of one biologic and the initiation of a second biologic.

^b^ From diagnosis to initiation of ustekinumab or vedolizumab

### Crohn’s disease outcomes

**FIGURE 1:**
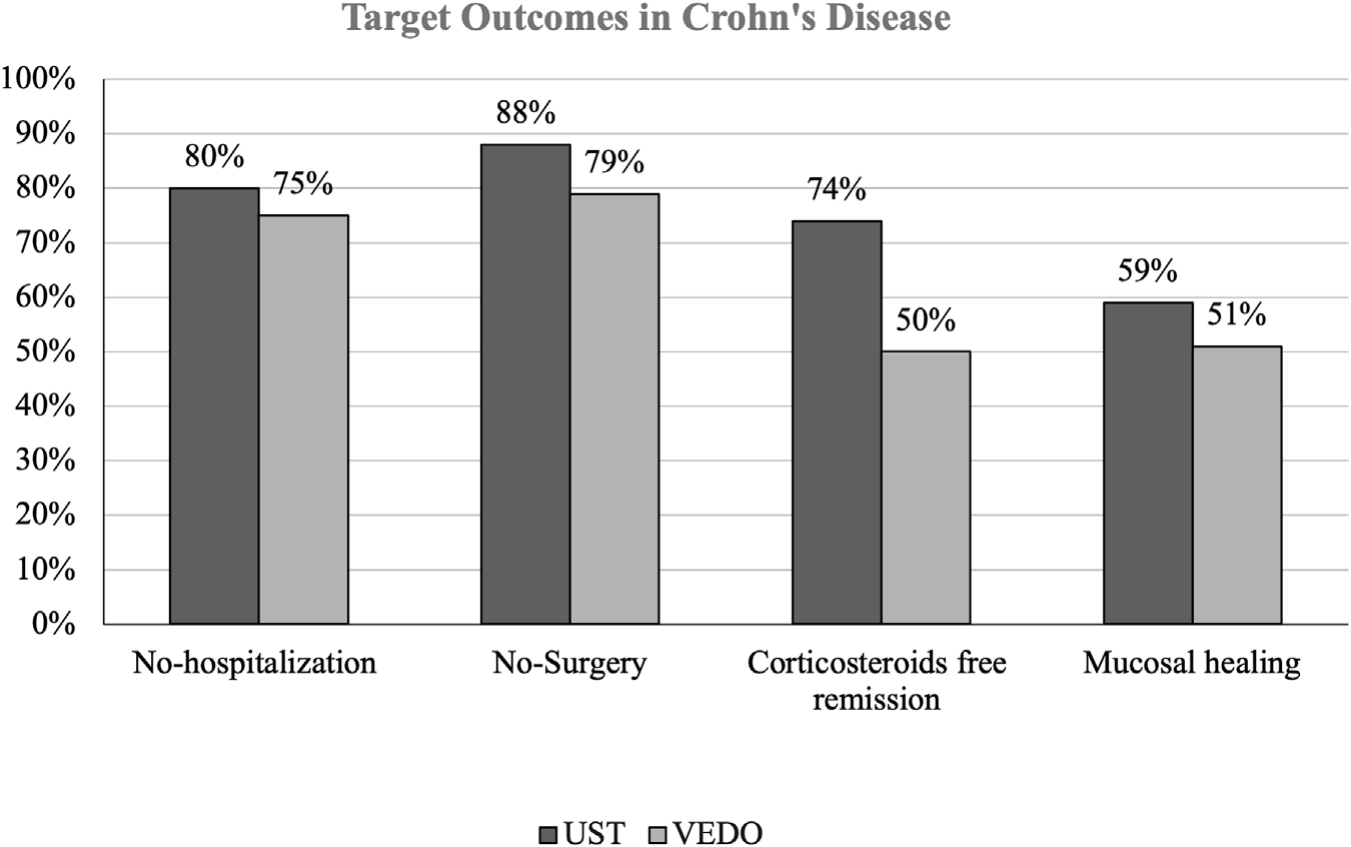
THIs figure shows the percentage difference in target outcomes between ustekinumab and vedolizumab in patients with Crohn’s disease (CD).

In patients with CD, 81 (62.5%) have failed infliximab, and 49 (37.5%) have failed adalimumab. 101 (77.6%) patients with CD who failed one anti-TNF therapy were on ustekinumab. Of these patients, 26 (19.7%) patients were hospitalized, and 12 (11.9%) patients had IBD-related surgery over 52 weeks. Regarding corticosteroid-free remission, 16 (16.1%) patients had at least one corticosteroid course. 60 (59.0%) patients with CD on ustekinumab achieved mucosal healing (Figure 1).

29 (22.3%) patients with CD who failed one anti-TNF therapy were receiving vedolizumab. Of those, 7 (25%) patients were hospitalized, and 11 (37.9%) patients had IBD-related surgery. Furthermore, 14 (50%) patients had at least 1 corticosteroid course and 15 (51.0%) patients achieved mucosal healing. Among the 12 patients who had IBD-related surgery, majority of patients on ustekinumab had small bowel resection while only 2 patients had small bowel resection with right hemicolectomy. Similar findings were observed in patients taking vedolizumab, 11 patients had small bowel resection, and none had small bowel resection with right hemicolectomy (Table 3).

**TABLE 3:** Patients with Crohn’s disease who had IBD-related surgery.

	Small bowel resection	Small bowel resection + right hemicolectomy
Ustekinumab (*n* = 12)	10	2
Vedolizumab (*n* = 11)	11	0

Subgroup analysis in patients with CD receiving ustekinumab, showed that 6 out of the 26 hospitalized patients were admitted more than once for IBD related causes, while 9 out of 16 patients received 2 or more courses of corticosteroids.

### Ulcerative colitis outcomes

**FIGURE 2:**
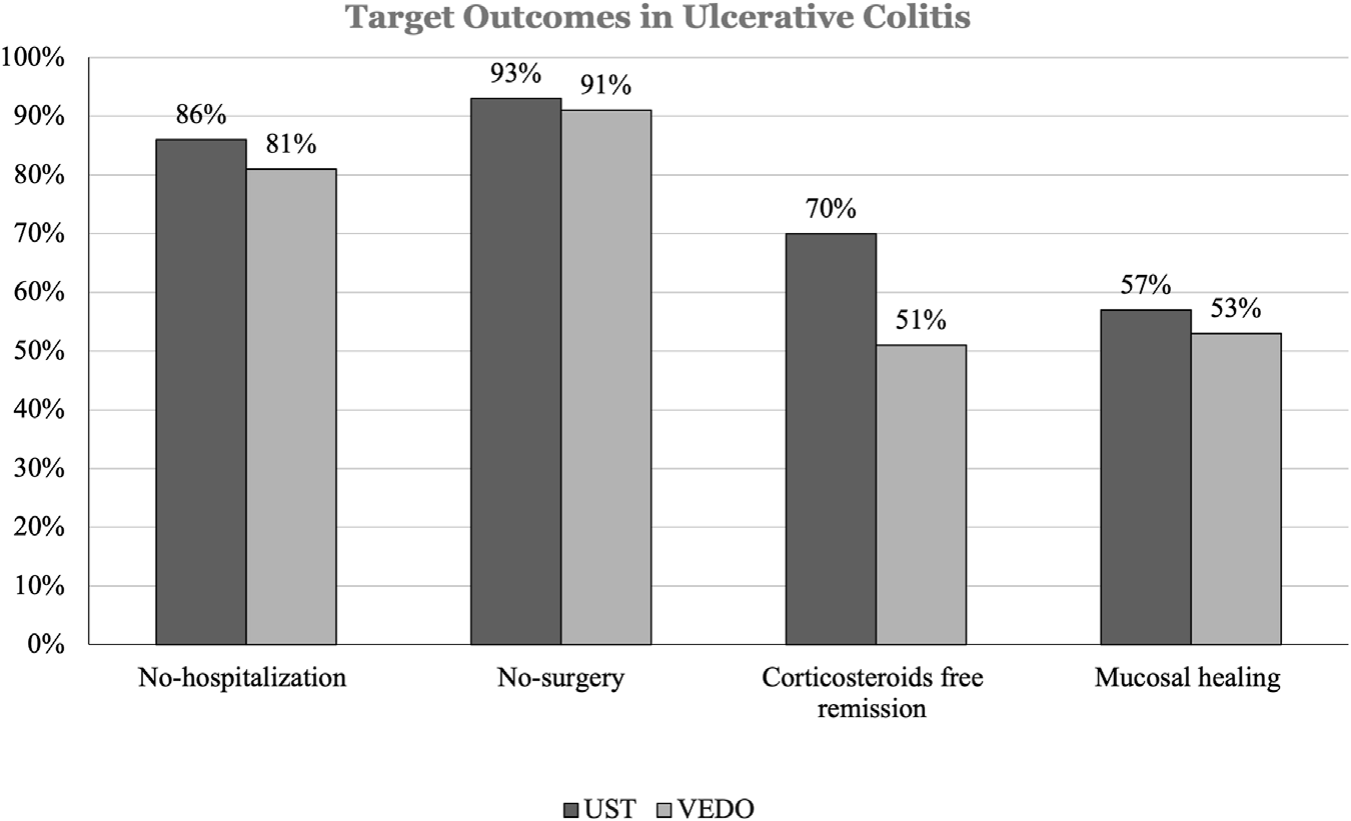
This figure shows the percentage difference in target outcomes between ustekinumab and vedolizumab in patients with ulcerative colitis (UC).

In patients with UC, 42 (54.5%) have failed infliximab, and 35 (45.5%) have failed adalimumab. 44 (57.1%) patients with UC who failed one anti-TNF therapy were on ustekinumab. Of these 6 (14.1%) patients were hospitalized and 3 (7.0%) patients had IBD-related surgery over 52 weeks. In addition, 13 (30%) patients had at least 1 corticosteroid course. 25 (57.0%) patients achieved mucosal healing (Figure 2).

33 (42.8%) patients with UC who failed one anti-TNF therapy were receiving vedolizumab. Of those, 6 (18.6%) patients were hospitalized, and 3 (9.0%) patients had IBD-related surgery over 52 weeks. Furthermore, 16 (49.6%) patients had at least 1 corticosteroid course. 17 (53.2%) patients achieved mucosal healing. Among the 3 patients who had IBD-related surgery, all patients on ustekinumab had colectomy followed by Ileal Pouch Anal Anastomosis (IPAA). Similar findings were observed in patients taking vedolizumab, all 3 patients had colectomy followed by IPAA, and none had proctocolectomy with end ileostomy (Table 4).

**TABLE 4:** Patients with ulcerative colitis who had IBD-related surgery.

	Proctocolectomy with end ileostomy	Colectomy followed by IPAA
Ustekinumab (*n* = 3)	0	3
Vedolizumab (*n* = 3)	0	3

IPAA: ileal pouch anal anastomosis.

Subgroup analysis in patients with UC receiving ustekinumab, showed that 3 out of 13 patients received 2 or more courses of corticosteroids. While subgroup analysis in patients with UC receiving vedolizumab showed that 2 out of 16 patients had 2 or more courses of corticosteroids. In terms of IBD-related hospitalization, all included patients with UC were admitted only once as described above.

## Discussion

This study evaluated the effectiveness of ustekinumab and vedolizumab in a cohort of patients with IBD who had previously received at least one anti-TNF agent. The primary outcomes were the percentage of hospitalization, surgery, steroid-free remission, and mucosal healing, defined as Mayo score of 0 or 1 in UC and a SES-CD score of less than 3 in CD. Numerically, patients receiving ustekinumab had better clinical target outcomes than patients receiving vedolizumab after failing anti-TNF therapy. However, both agents showed effectiveness in patients with IBD after failing anti-TNF therapy.

During the GEMINI trial, vedolizumab has shown efficacy in both anti-TNF experienced and anti-TNF-naïve patients with UC (Feagan et al., 2013). A multi-center retrospective study compared the efficacy and safety of vedolizumab and infliximab in patients with UC after failing an anti-TNF agent (Hupé et al., 2020). The study included 225 patients and the authors found that compared with 26% of patients treated with infliximab, 49% of patients treated with vedolizumab as their second-line agent achieved clinical remission after a median duration of 14 weeks (*p* < 0.01). However, *Post hoc* analyses from the GEMINI 2 and GEMINI 3 trials showed that at weeks 6 and 10, clinical response rates were numerically higher in the anti-TNF-naïve patients with CD [40.3% and 48.4%] in comparison to the anti-TNF-exposed group [33.1% and 39.7%], respectively (Sands et al., 2017). Differences in clinical response rate persisted throughout week 52. Similar findings were observed in patients with UC using the *post hoc* analysis of efficacy data from the GEMINI 1 study. The *post hoc* analysis included 464 TNF-naïve patients and 367 TNF-failure and at week 6 clinical response rates to vedolizumab were numerically higher in TNF-naïve patients compared to patients who had failed anti-TNF previously (Feagan et al., 2017).

One study (Rayer et al., 2022) performed in France included patients with CD who failed a first anti-TNF drug, and treated with a second anti-TNF agent, ustekinumab, or vedolizumab as a second-line biological therapy. The authors found that the rates of steroid-free remission at weeks 14–24 were similar between the ustekinumab, vedolizumab, and second anti-TNF groups (29%, 38%, and 44%, respectively, *p* = 0.15). The study concluded that in the short term ustekinumab, vedolizumab, and a second anti-TNF agent demonstrated similar efficacy, as second-biological line treatment in patients with CD after failure of an initial anti-TNF agent. Another study examined the efficacy of ustekinumab in patients with refractory CD who have failed an anti-TNF agent or vedolizumab (Verstockt et al., 2019). After 24 weeks, the study found that ustekinumab showed good clinical remission rates (39.5%) but only 7% of the cohort achieved endoscopic remission.

A retrospective study examined the real-world effectiveness and safety outcomes of vedolizumab in patients with UC who had failed anti-TNF therapy in Korea (Ye et al., 2021). The study included 105 patients and authors found that within the first 14 weeks of use, vedolizumab is effective and well tolerated in the real-world setting.

Evidence from the Literature suggests an absence of difference between vedolizumab and ustekinumab after failing one anti-TNF agent in patients with CD (Pagnini et al., 2018; Singh et al., 2018; Alric et al., 2020). However, two studies favored using ustekinumab over vedolizumab because long-term remission was significantly higher in the ustekinumab group (Alric et al., 2020; Biemans et al., 2020).

A study suggested that a high proportion of patients continued on their initial biologics for 1 year and approximately half of patients were persistent after 5 years of treatment (Barbieri et al., 2022). The same study, found that patients treated with vedolizumab and ustekinumab seemed to have a higher risk of non-persistence compared to patients treated with infliximab. Authors attributed this non-persistence to be related to age and gender, in addition to those who switch/swap and those who experienced ADRs.

This study has important clinical implications. Guidance on the most appropriate second-line therapy after failing an anti-TNF agent is sparse. This study adds to evidence regarding the effectiveness of ustekinumab and vedolizumab after anti-TNF failure. Furthermore, with the increasing numbers of emerging novel therapies for IBD, it is essential to understand the best sequence of treatments in patients who have failed at least one anti-TNF agent. Randomly selecting second-line therapies for the treatment of IBD patients may lead to delays in achieving clinical response and remission.

To the best of our knowledge, this is the first study in Kuwait and middle east region to assess the efficacy of vedolizumab and ustekinumab after failing anti-TNF therapy. Additionally, the use of clinically relevant endpoints strengthens the applicability and clinical value of this study. In addition, endoscopic remission was also included as a long-term outcome which is recommended by STRIDE II guidelines (Turner et al., 2021). Furthermore, the strict inclusion criteria aid in the precise assessment of the efficacy of the second-line biologic. Moreover, long follow-up period (52 weeks) help in accurately assessing the efficacy of treatment with ustekinumab, and vedolizumab in IBD after failing anti-TNF therapy.

However, this study is not without limitations. It is a retrospective, single-center study therefore, generalization potential is limited. Furthermore, similar real world studies in Europe has been published before (Alric et al., 2020; Hupé et al., 2020; Onali et al., 2022; Rayer et al., 2022; Straatmijer et al., 2023). Additionally, given the observational nature of the present study, confounding effects cannot be completely eliminated, potential confounding factors remain a possible limitation of this study. Finally, comparison between effectiveness of vedolizumab and ustekinumab was not possible because of the small number of study subjects.

In conclusion, ustekinumab and vedolizumab were both effective in achieving clinical outcomes in patients with IBD after failing anti-TNF therapy. However, patients receiving ustekinumab had numerically higher percentages of reaching target outcomes than patients receiving vedolizumab. A prospective head-to-head trial is warranted to confirm these findings. Direct head-to-head active comparator trials, exclusively in patients with previous biologic exposure are needed in order to determine most appropriate second line therapy.

## Data Availability

The raw data supporting the conclusion of this article will be made available by the authors, without undue reservation.
